# Label-Free Liquid Chromatography–Mass Spectrometry Proteomic Analysis of Urinary Identification in Diabetic Vascular Dementia in a Han Chinese Population

**DOI:** 10.3389/fnagi.2021.619945

**Published:** 2021-02-01

**Authors:** Ruijuan Chen, Yuanjing Yi, Wenbiao Xiao, Bowen Zhong, Yi Shu, Le Zhang, Yi Zeng

**Affiliations:** ^1^Department of Geriatrics, Second Xiangya Hospital, Central South University, Changsha, China; ^2^Department of Neurology, Xiangya Hospital, Central South University, Changsha, China; ^3^State Key Laboratory of Proteomics, Beijing Proteome Research Center, National Center for Protein Sciences (Beijing), Beijing Institute of Lifeomics, Beijing, China; ^4^Department of Neurology, Second Xiangya Hospital, Central South University, Changsha, China

**Keywords:** diabetes, vascular dementia, biomarkers, protein, urine, proteomics

## Abstract

**Objective:** This study aimed to identify potential diagnostic biomarkers of diabetic vascular dementia (DVD) and unravel the underlying mechanisms using mass spectrometry (MS).

**Methods:** Label-free liquid chromatography-tandem mass spectrometry (LC-MS/MS) proteomic analysis was applied to urine samples from four groups, including 14 patients with vascular dementia (VD), 22 patients with type 2 diabetes mellitus (T2DM), 12 patients with DVD, and 21 normal controls (NCs). Searching the MS data by Proteome Discoverer software (ThermoFisher Scientific; Waltham, MA, USA), protein abundances were analyzed qualitatively and quantitatively and compared between these groups. Combining bioinformatics analysis using Gene Ontology (GO), pathway crosstalk analysis using Kyoto Encyclopedia of Genes and Genomes (KEGG), protein–protein interaction (PPI) network analysis using STRING, and literature searching, the differentially expressed proteins (DEPs) of DVD can be comprehensively judged and were further quantified by receiver operating characteristic (ROC) curve methods.

**Results:** The proteomic findings showed quantitative changes in patients with DVD compared to patients with NC, T2DM, and VD groups; among 4,744 identified urine proteins, 1,222, 1,152, and 1,180 proteins displayed quantitative changes unique to DVD vs. NC, T2DM, and VD, respectively, including 481 overlapped common DEPs. Then, nine unique proteins [including HP, SERPIND, ATP5PB, VNN2, ALDH3A1, U2AF2, C6, A0A5C2GRG5 (no name), and A0A5C2FZ29 (no name)] and two composite markers (CM) (A0A5C2GRG5+U2AF2 and U2AF2+C6) were confirmed by a ROC curve method.

**Conclusion:** This study provided an insight into the potential pathogenesis of DVD and elucidated a method for early detection.

## Introduction

Dementia, a disabling condition with functional impairment in language, memory, and execution functions, currently affects nearly 50 million people worldwide (World Health Organization, 2017). According to the World Alzheimer Report 2015, the number was expected to increase to 75.6 million by 2030 and more than 130 million by 2050 (Prina et al., 2015). Vascular dementia (VD) is one of the most common types of dementia, accounting for about 20% of all dementia cases (Hugo and Ganguli, 2014; O'Brien and Thomas, 2015). Similarly, type 2 diabetes mellitus (T2DM) is one of the most common type of chronic metabolic diseases characterized by insulin resistance and relative insulin deficiency, accounting for about 90% of DM, which affects more than 300 million people worldwide (Reiman, 2014). Globally, due to the aging population, they together have become a serious public health burden with significant financial and social implications. Expanding epidemiological data showed that DM was associated with VD, which can increase the risk of dementia by two to three times, and was even considered to be a major contributory factor for dementia in the elderly population (Cheng et al., 2012; Biessels et al., 2014). Diabetes was proved to be a well-established risk factor for microvascular and macrovascular complications, such as stroke (Peters et al., 2014), which also suggests that there is a robust association between DM and VD. However, the underlying pathophysiological mechanisms for this close association remain unclear. A multifactorial pathogenesis that involves cerebrovascular damage, insulin metabolism, hyperglycemic toxicity, and chronic inflammation may contribute to this biological relationship, which seems to be treatable and reversible (Ahtiluoto et al., 2010; Ninomiya, 2014). Therefore, early diagnosis provides a great possibility for the therapeutic intervention of VD caused by DM.

To date, there is no non-invasive quantitative biological marker for an early diagnosis of diabetic VD (DVD), which only depends on the medical history, various neuropsychological scales, clinical laboratory tests, or highly expensive imaging examinations, such as β-amyloid peptide-PET (Aβ-PET). Biomarkers, which can reflect the pathological or physiological changes of disease, have been applied to facilitate disease diagnosis such as cancer (Capello et al., 2017). The application of proteomics in cerebrospinal fluid (CSF) and plasma have significantly accelerated the unbiased and high-throughput search for potential biomarkers of neurodegenerative disorders, albeit limited in Alzheimer's disease (AD) or other types of dementia without DM (Rosa-Neto et al., 2013). Since urine is not affected by homeostasis mechanisms, accumulates a large number of changes in the blood (An and Gao, 2015), and is more convenient compared with CSF obtained through an invasive procedure, urine can be used as a potential source of disease biomarkers to replace blood or CSF. However, to date, urinary biomarkers used for brain diseases are mostly ignored due to the distance between the brain and the urinary tract, leading to less research on urinary biomarkers in dementia.

This study is specifically designed to obtain different insights into VD with the discovery of the DM biomarker, focusing on urinary protein biomarkers. Urinary samples from patients with DVD, VD, DM, and normal controls (NCs) were analyzed using the label-free high-performance liquid chromatography-tandem mass spectrometry (LC-MS/MS; HPLC-MS/MS). Differentially expressed proteins (DEPs) identified by the proteomic analysis were further quantified both graphically and statistically with receiver operating characteristic (ROC) curve methods (Abdi et al., 2006). Besides, two top composite markers (CM) have been identified as potential biomarkers or DVD-associated proteins, which were also validated *via* ROC curves in the current study.

## Materials and Methods

### Ethics Approval

The research protocol was approved by the Ethics Committee of the Second Xiangya Hospital of Central South University, and written informed consent was obtained from participants following the Declaration of Helsinki and the independent ethics committee or institutional review board.

### Study Design and Population

**Figure 1:**
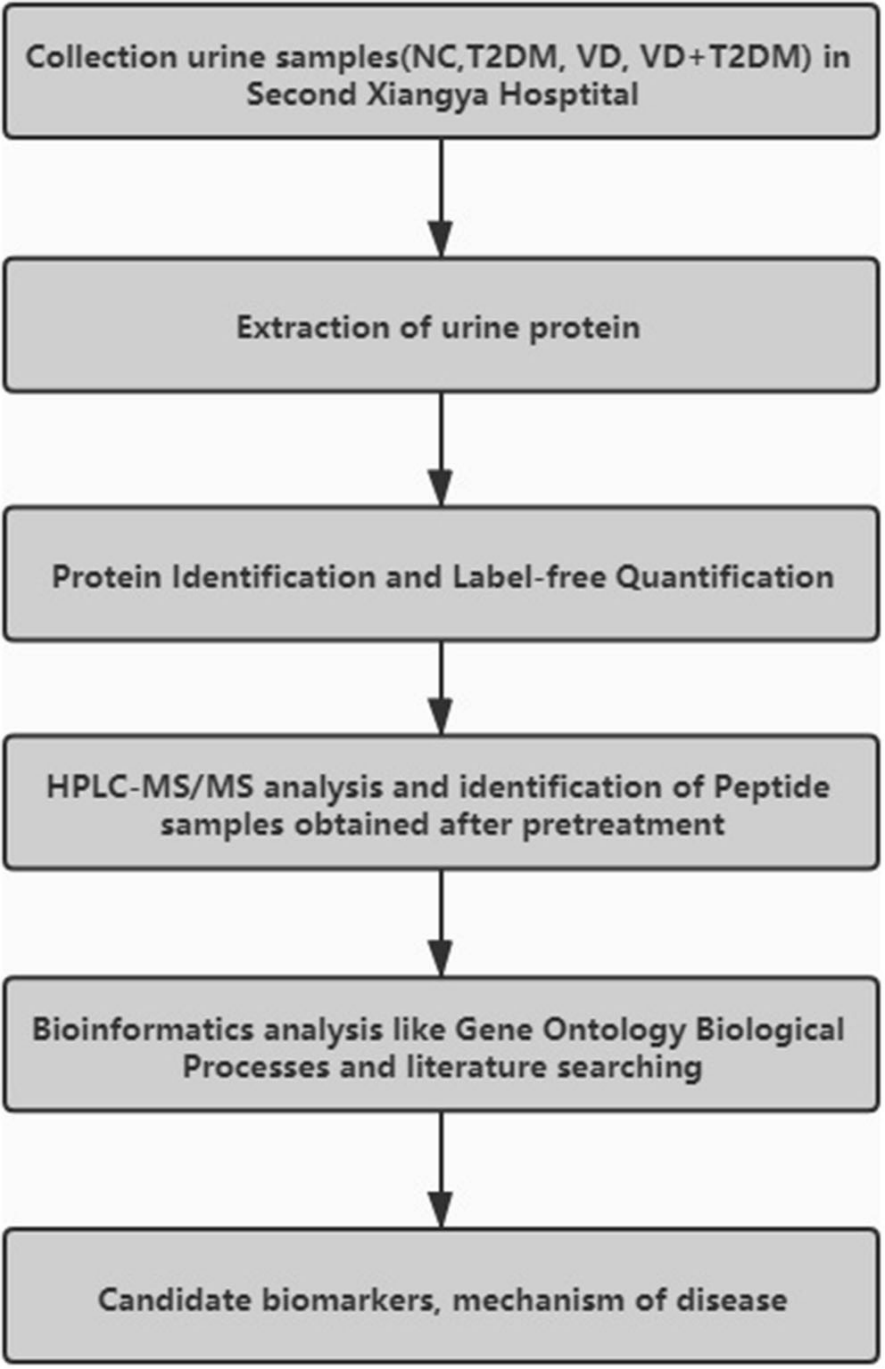
The workflow of the experimental procedures.

The workflow of this study is given below in Figure 1. A total of 69 subjects were enrolled in the Second Xiangya Hospital of Central South University from 2016 to 2017, including 14 subjects with VD, 22 subjects with T2DM, 12 subjects with DVD (or VD+T2DM), 21 NC patients matched with their age and sex. All NC patients were recruited from the Health Examination Center of the Second Xiangya Hospital during the same period, with no history of chronic disease.

### Inclusion/Exclusion Criteria and Clinical Examination

All patients with VDwere diagnosed by neuropsychiatrists in the hospitals based on the criteria defined by the National Institute of Neurological Disorders and Stroke-Association Internationale pour la Recherche et l'Enseignement en Neurosciences (NINDS-AIREN) (Román et al., 1993; Pohjasvaara et al., 2000) and the central assessment of the neuroimaging criteria for VD. Patients with VD had to fulfill a Mini-Mental Status Examination (MMSE). Patients with an MMSE score of 10–24 were included in the research. All subjects with VD must have a history of stroke of at least 3 months earlier attributed to large artery atherosclerosis, small artery occlusion, cardioembolism, or unidentified/other.

Exclusion criteria include: (1) patients with a history of stroke within 3 months prior to baseline unless the function of the patient has been fully stabilized; (2) a current diagnosis of any primary neurodegenerative disorder, such as AD using the Hachinski Ischemia Score (HIS) scale; (3) a current diagnosis of major depression using the Hamilton Depression Rating Scale (HAMD) and the Hamilton Anxiety Rating Scale (HAMA); (4) patients with lobar hemorrhages or space-occupying lesions. The diagnosis of T2DM was based on the WHO criteria (Boulton, 1998); (5) patients with type 1 DM (T1DM) or any specific types of diabetes other than T2DM; (6) patients with episodes of severe hypoglycemia; and (7) patients with acute or chronic complications of T2DM.

Patients with a history of T2DM or abnormalities in random blood glucose or fasting blood glucose for at least 1 year before the stroke were classified as the T2DM group, while those combined with VD were classified as the VD+T2DM group. The age of the participants ranged from 71 to 88 years. The male to female ratio was about 2:1. All participants are of ethnic Han Chinese origin.

### Urinary Sample Collection and Processing

About 10–20 ml of midstream specimens of the first-morning urine was collected to establish the standard operation procedure (SOP) and centrifuged at 2,000 r/min for 10 min. Then, the cells and cell debris were removed and the supernatant was retained, separated, and stored at −80°C. Urinary proteome data were analyzed by using LC-MS/MS (Leng et al., 2017; Gao, 2019).

### Protein Precipitation, Quantification, and In-Solution Trypsin Digestion

One milliliter of urinary sample reheated at 37°C was centrifuged at 4°C, 176,000 g for 70 min to discard the supernatant and retain the precipitation. Next, 40 μl of resuspension buffer (50 mM Tris, 250 mM sucrose, pH 8.5) was added, and the urinary protein was resuspended in the buffer solution after standing for 10 min. Then, 2.5 μl 1 mol/L dithiothreitol (DTT) was added and heated at 65°C for 30 min to decompose the protein into a polypeptide. The second ultracentrifugation was processed for 40 min after adding the wash buffer (10 mM TEA, 100 mM NaCl, pH 7.4). Thirty microliters of the prepared digestion buffer (50 mM NH_4_CO_3_) was added and heated at 95°C for 3 min to cool down to room temperature. Ten microliters of 100 ng/μl trypsin (Promega, USA) MS grade was added, and the protein was digested overnight at 37°C. Finally, 100 μl of acetonitrile (ACN, Sigma, USA) was added, oscillated for 5 min, centrifuged at 10,000 g for 5 min, and the supernatant was aspirated and vacuum dried for mass spectrometer detection.

### Mass Spectrometry Analysis and Protein Identification

A total of 69 samples from four groups (NC, T2DM, VD, and VD+T2DM groups) were analyzed by using a Q Exactive HF quadrupole-Orbitrap mass spectrometer (Thermo Fisher Scientific) coupled to UltiMate 3000 HPLC and UHPLC Systems (Thermo Fisher Scientific). Each digested-peptide sample (5 μl) was redissolved for nanoscale LC-MS/MS (nano-LC-MS/MS) analysis and loaded onto a reversed-phase C18 self-packed capillary LC column (1.7 μm, 120 Å, 150 μm × 12 cm, Dr. Maisch, Germany) with two solvent buffer (A: 99.9% water and 0.1% formic acid; B: 79.9% ACN, 20% water, and 0.1% formic acid) for 75 min non-linear gradient of 6–35% ACN at a flow rate of 600 Nl/min. Peptides were analyzed by data-dependent MS/MS acquisition mode with a resolution of 60,000 at a full scan mode and 15,000 at the MS/MS mode. The full scan was processed in the Orbitrap from 300 to 1,400 m/z; the top 30 most intense ions in each scan were automatically selected for high-energy collision dissociation (HCD) fragmentation with a normalized collision energy of 27% and measured in the Orbitrap. Typical mass spectrometric conditions contained: Automatic gain control (AGC) targets were 3 × e6 ions for full scans and 1 × e5 for MS/MS scans; the maximum injection time was 80 ms for full scans and 40 ms for MS/MS scans, and dynamic exclusion was employed for 15 s. Proteins were identified and quantified against the complete human proteins in the Uniprot database (2020.07.02) using Proteome Discover 2.4 software (Thermo Fisher Scientific) with SEQUEST and Mascot search engine (version 2.3.01, Matrix Science, London, UK). The parameters were set as follows: A maximum of two missed cleavage was allowed; Carbamido methylation of cysteines was considered as a fixed modification, and the oxidation of methionine and protein N-terminal acetylation was classified as variable modifications. We retained a false discovery rate (FDR) of <1% and with at least two unique peptides to identify and quantify proteins. Then, Spectral Counting Tandem MS was explored using the SEQUEST algorithm in BioworksBrowser (v3.31, ThermoFisher Scientific; Waltham, MA, USA; Eng et al., 1994), and protein quantification was calculated using “TOP THREE ANALYSIS” (the first three high-intensity peptides were quantified and determined for each protein) as described in a study of Krey et al. (2014). All missing values of protein in the sample were substituted with zero. Besides, when compared with two groups, if the protein expression in more than half of the samples in one group is zero, and the protein expression in more than half of the samples in the other group is non-zero, it means that the protein is not expressed in this group and belongs to another group-specific protein, the value of *p* will be designated as 0, which is significant. If this protein is expressed in more than 50% of the samples in both groups, the differences are compared using the two-tailed *t*-test (*p* < *0.05*). To ensure accurate quantification, proteins identified in each sample along two technical replicates were normalized to the summed intensity.

### Bioinformatics and Statistical Analysis

*T*-tests comparing the sample groups using log_2_ transformed ratios were used to determine whether proteins were differentially accumulated (*p* < 0.05 and *FC* < 0.83 or > 1.20). Interpersonal coefficients of variation (CVs) were calculated for all datasets using only proteins abundant with non-zero quantification values. The CV is equal to mean/SD. The proteins were further elucidated for functional classifications according to the Gene Ontology (GO; http://www.geneontology.org) project and the Kyoto Encyclopedia of Genes and Genomes (KEGG) pathway enrichment analysis (https://www.kegg.jp/kegg/genes.html) using GOATOOLS (v 0.6.5) (Jensen et al., 2009) and KOBAS (v2.1.1) software. Fisher's exact test was used in these analyses. To control the calculation of false positives, GOATOOLS provided multiple test methods to correct the values of *p*, such as Bonferroni's, Holm's, Benjamini and Hochberg's (BH), and Benjamini and Yekutieli's (BY) tests. Under normal circumstances, the value of p was corrected by default using the BH method. When the corrected value of *p* (FDR) is < *0.05*, it was considered that there is a significant enrichment. The protein–protein interaction (PPI) networks associated with these proteins were further performed using (Search Tool for the Retrieval of Interacting Genes/Proteins) STRING DB v11 (https://string-db.org/cgi/network.pl) and Cytoscape (v3.6.1). R Studio and SPSS 25.0 statistical software were used for data statistical analysis. The data were presented as mean ± SEM, and statistical analyses were performed by the two-tailed *t*-test and one-way ANOVA for the comparison between groups; *p* < 0.05 was considered as significantly different in the protein expression between the DVD and other three groups separately.

### Evaluating the Diagnostic Ability of Candidate Markers

#### Evaluating the Diagnostic Ability of a Single Marker

The performance of single confirmed candidate markers screened by the proteomic analysis, which can be expressed in each group, was qualified both graphically and statistically with the ROC curve method between the DVD group and other three groups (as a whole group; Abdi et al., 2006). Wilcoxon Rank-Sum test was used to establish the statistical significance of a single marker and evaluate the significance of the whole ROC curve.

#### Composite Markers

After the selected candidate markers were qualified based on the sensitivity at 95% specificity, *p*-value, and the area under the curve (AUC) value, the significant individual markers were selected for calculation of a CM, which aggregates two markers into a diagnostic marker accomplished by evaluating a linear combination using logistic regression (Abdi et al., 2006). After the significance was established, we reexamined the obtained ROC curve to evaluate the quality of compound markers.

## Results

### Baseline Characteristics

Demographic and clinical characteristics of the study from subjects of NC, T2DM, VD, and VD+T2DM groups are presented in Table 1. There were no significant differences in age and years of education between the VD + T2DM, VD, T2DM, and NC groups. The majority of patients of the VD and VD+T2DM groups had a similar clinical profile: the history of hypertension; high rates of atherosclerosis; and stroke/transient ischemic attacks at least 3 months before; a significantly lower score of MMSE, clock-drawing test (CDT), and verbal category fluency test (VCFT); and a higher score of HAMA. All assessments of MMSE, CDT, VCFT, HAMD, and HAMA of T2DM and NC groups were normal. Compared with the other two groups, VD+T2DM and T2DM groups showed a slight increase in serum creatinine (CRE).

**Table 1:** Demographic and clinical characteristics of subjects and the numbers of samples for the NC, VD, T2DM, VD+T2DM groups.

	**VD + T2DM (*n* = 12)**	**VD (*n* = 14)**	**T2DM (*n* = 22)**	**NC (*n* = 21)**
Sex (female/male)	4/8	5/9	8/14	7/14
Age (year)	78.10 ± 10.48	76.00 ± 8.03	71.30 ± 8.57	72.67 ± 8.99
Years of education	8.30 ± 1.25	8.50 ± 2.11	8.15 ± 1.27	7.33 ± 1.27
History of hypertension (yes/no)	7/5	7/7	6/16	4/17
History of cardiovascular disease (yes/no)	5/7	6/8	3/18	2/19
History stroke/transient ischemic attacks for at least 3 months	12/12	14/14	0/22	0/21
CRE (μmol/l)	115.35 ± 55.17^**^	68.03 ± 16.68	97.05 ± 42.33^*^	71.11 ± 24.43
MMSE score	14.7 ± 2.26^**^	15.92 ± 4.39^**^	29.05 ± 1.00	29.4 ± 0.55
CDT score	1.40 ± 0.52^**^	1.25 ± 0.97^**^	4.00 ± 0.00	4.00 ± 0.00
VCFT score	7.90 ± 1.79^**^	7.50 ± 3.03^**^	14.15 ± 3.20	15.40 ± 0.89
HAMD score	5.90 ± 3.45	6.67 ± 1.61	5.35 ± 2.13	6.20 ± 1.30
HAMA score	8.40 ± 2.63^**^	8.08 ± 3.63^**^	3.25 ± 1.12	2.80 ± 1.48

* *Significant differences compared with the NC group at p < 0.05*;

** *Significant differences compared with the NC group at p < 0.01; CRE, serum creatinine; MMSE, Mini-Mental State Examination; CDT, Clock-Drawing Test; VCFT, Verbal Category Fluency Test; HAMD, Hamilton Depression Scale; HAMA, Hamilton Anxiety Scale*.

### Proteomics Findings

**Figure 2:**
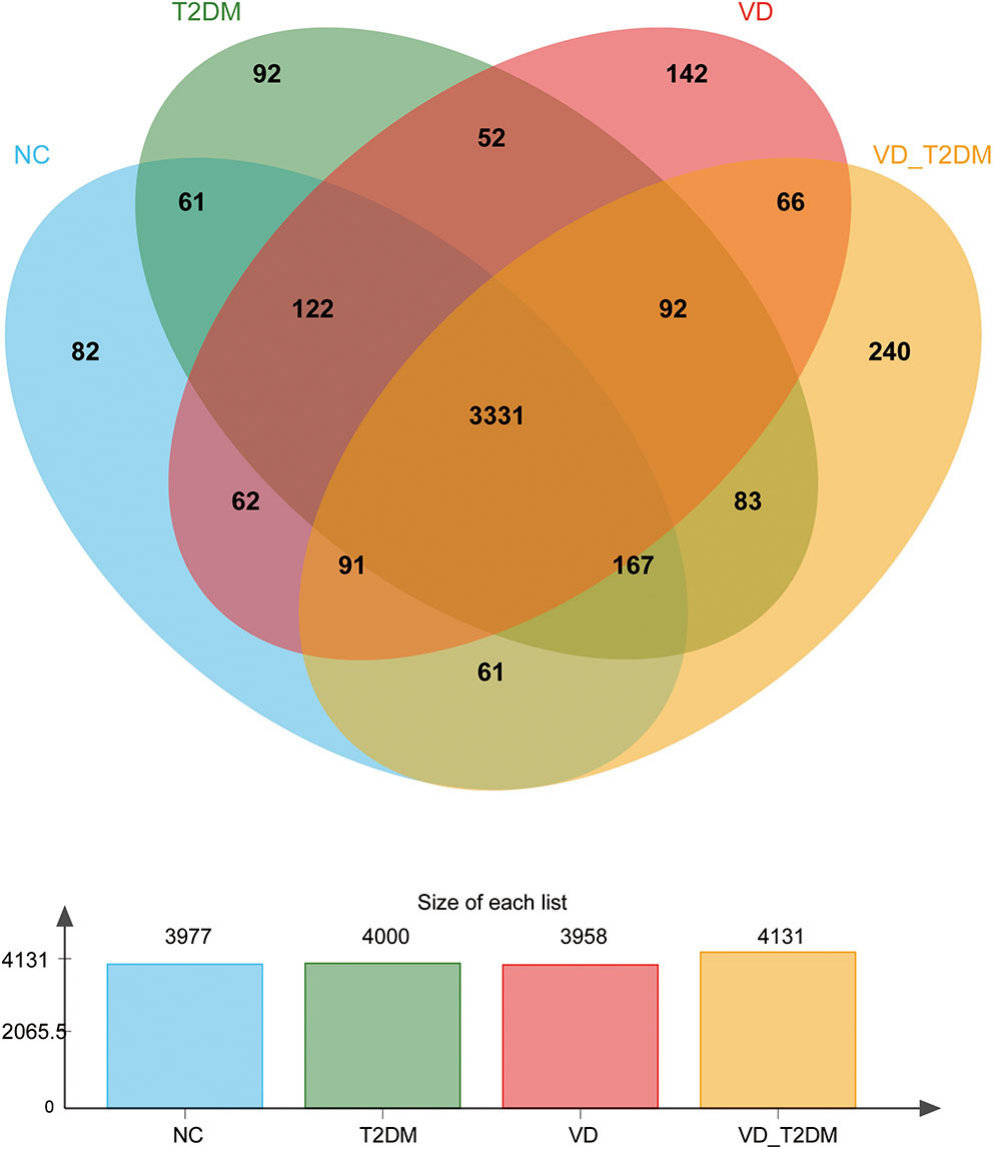
A Venn diagram illustration of the urinary proteins identified in normal controls (NC), type 2 diabetes mellitus (T2DM), vascular dementia (VD), and VD+T2DM groups. Representative Venn diagrams show common proteins found in pooled urine-enriched fractions from patients with DM+VD (*n* = 10), patients with VD (*n* = 12), patients with DM (*n* = 20), and normal controls (*n* = 21).

A total of 4,744 urinary proteins were successfully identified (Supplementary Table 5), out of which 3,977, 4,000, 3,958, and 4,131 urinary proteins were identified in NC, T2DM, VD, and VD+T2DM groups, respectively. Comparing the proteins, 3,331 proteins were found to be common among the four groups (Figure 2), suggesting that they share the overlapping regulatory mechanism of protein molecules.

### Differential Proteins Identified

#### VD+T2DM vs. NC

**Figure 3:**
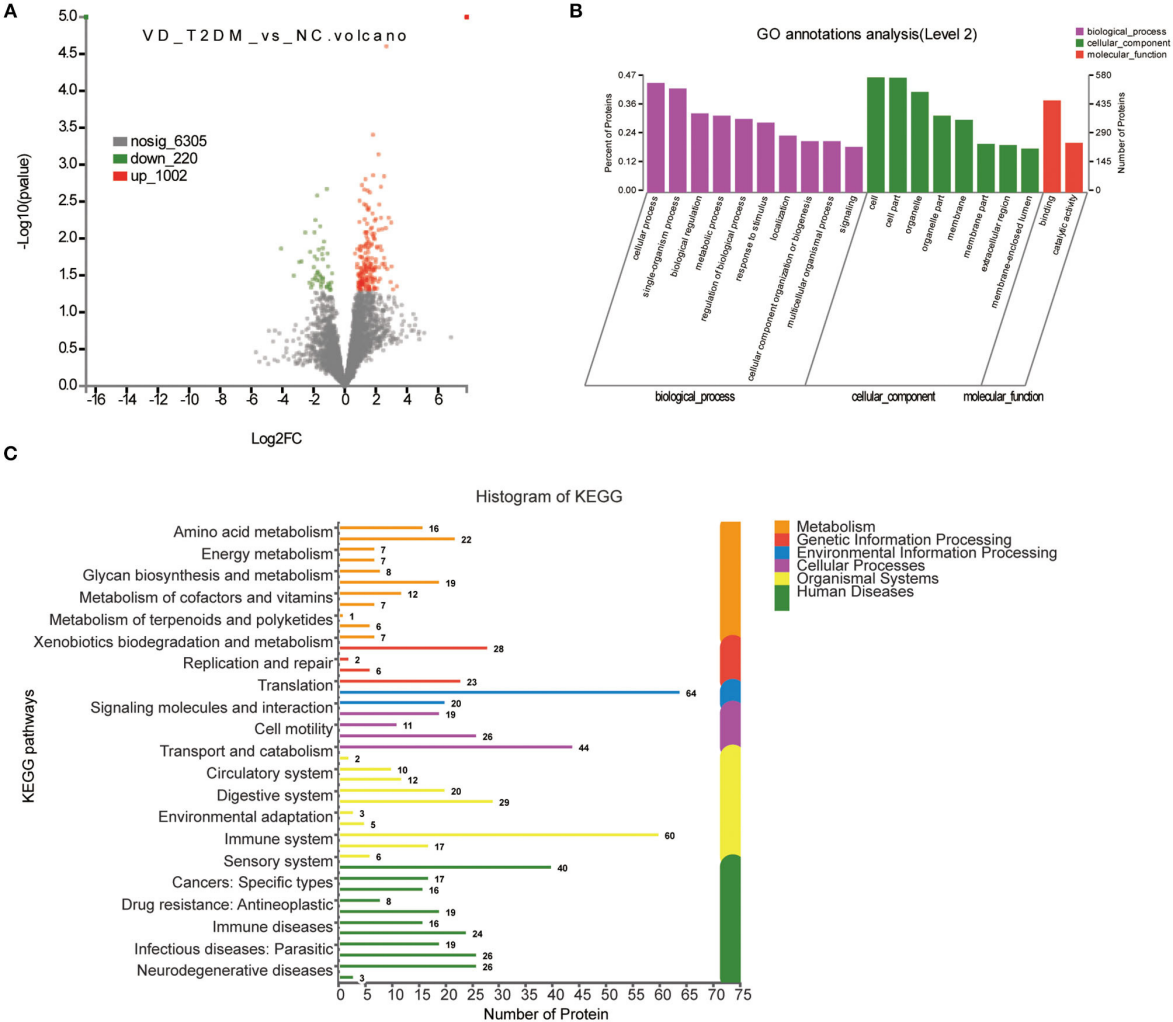
Volcano plots of quantified proteins and functional analysis of the differentially expressed proteins (DEPs) in the urine between subjects with VD+T2DM and NCs. **(A)** Volcano plot of mean TMT log_2_ ratio plotted against –log_10_ (*p*-value) of the one-sample *t*-test for the respective quantified proteins. Each point in the figure represents a specific protein. The point on the left is the protein with a downregulated expression, and the point on the right is the protein with an upregulated expression. Green points represent downregulated differential proteins and red points represent upregulated differential proteins. **(B)** Gene Ontology (GO) enrichment analysis of differential urine-excretory proteins. Purple bars denote the enriched biological processes, green bars denote enriched cellular components, and red bars denote enriched molecular functions. Protein numbers enriched in each category are presented with the bars. **(C)** The DEPs are associated with diseases and signal transduction pathways. The number of proteins associated with each category is listed. The different color pathways represent the different categories to which they belong. According to the KEGG metabolic pathways involved in proteins, they can be divided into seven categories: metabolism (M), genetic information processing (GIP), environmental information processing (EIP), cellular processes (CP), organismal systems (OS), human diseases (HD), and drug development (DD).

A total of 1,222 DEPs identified by VD+T2DM vs. NC, among which 220 upregulated genes and 1,002 downregulated genes were encoded, were predicted to be urinary excretion in two groups (Figure 3A). Details of these DEPs were provided in Supplementary Table 1. GO enrichment analyses were further conducted by using Blast2GO, which is a bioinformatics platform for high-quality protein function prediction and functional analysis of genomic datasets. Figure 3B shows the enriched GO terms (Level 2), such as biological processes, cellular components, and molecular functions. These proteins were mainly involved in biological processes, such as cellular process, single-organism process, biological regulation, and metabolic process; the regulation of the biological process, the response to the stimulus, localization, cellular component organization or biogenesis, and signaling. According to the KEGG metabolic pathways involved in proteins, they can be classified into seven categories: metabolism (M), genetic information processing (GIP), environmental information processing (EIP), cellular processes (CP), organismal systems (OS), human diseases (HD), and drug development (DD). In this comparison, the metabolic pathways to protein annotation mainly included amino acid metabolism, energy metabolism, the biosynthesis of glycans and metabolism, the metabolism of cofactors and vitamins, metabolism of terpenoids and polyketides, xenobiotic biodegradation and metabolism. Also, 26 proteins were annotated for neurodegenerative diseases (Figure 3C).

#### VD+T2DM vs. T2DM

**Figure 4:**
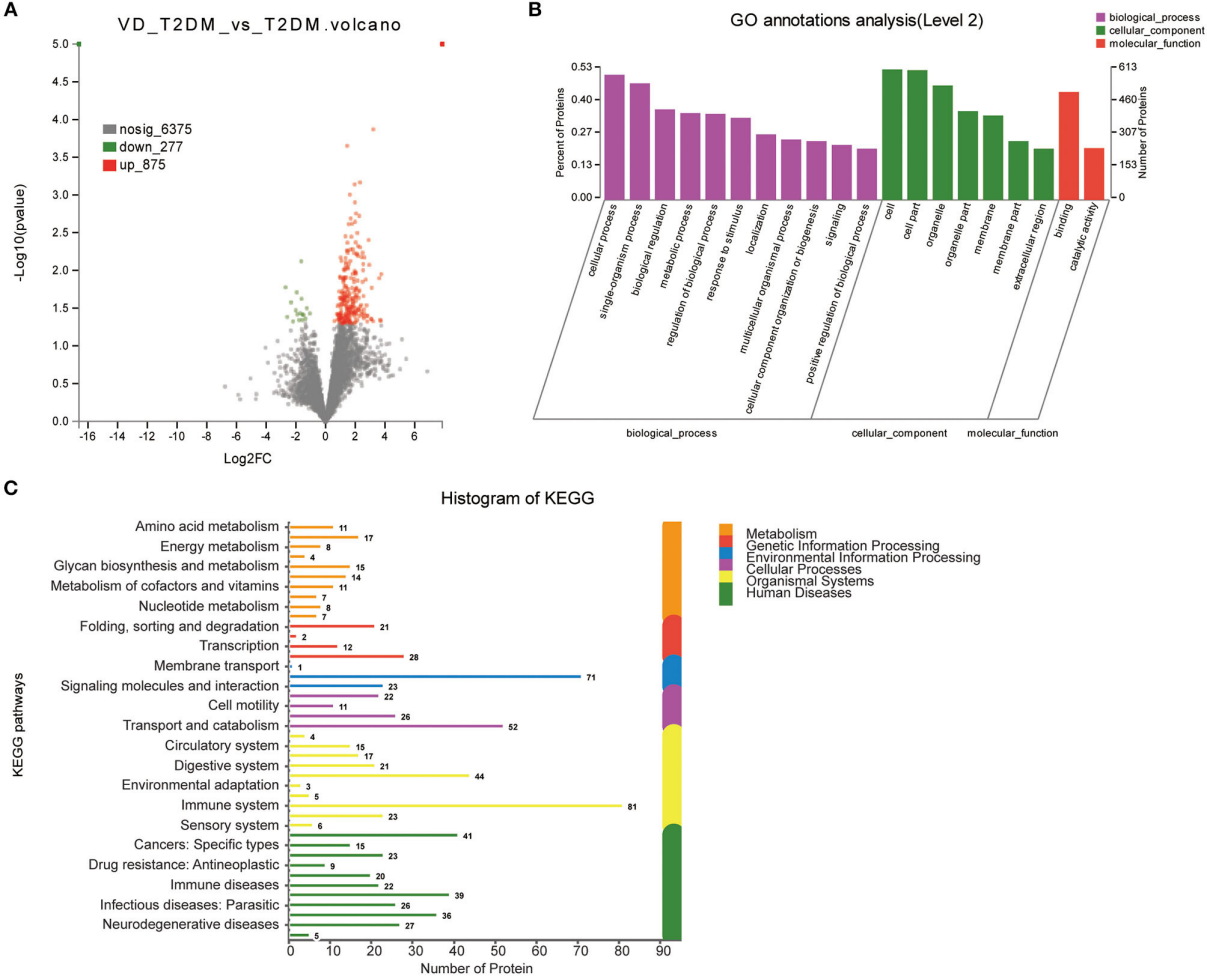
Volcano plots of quantified proteins and functional analysis of DEPs in the urine between subjects with VD+T2DM and T2DM (notes same as Figures 1–3).

Of all 1,152 DEPs identified by comparing VD+T2DM and T2DM subjects, 277 proteins were upregulated and 875 proteins were downregulated (Figure 4A). The main biological processes enriched GO terms were similar to VD+T2DM vs. NC, in addition to the positive regulation of biological processes (Figure 4B). Amino acid metabolism, energy metabolism, the biosynthesis of glycans and metabolism, the metabolism of cofactors and vitamins, and nucleotide metabolism were involved in the M pathway, and 27 proteins were associated with neurodegenerative diseases by the KEGG metabolic pathway analysis (Figure 4C).

#### VD+T2DM vs. VD

**Figure 5:**
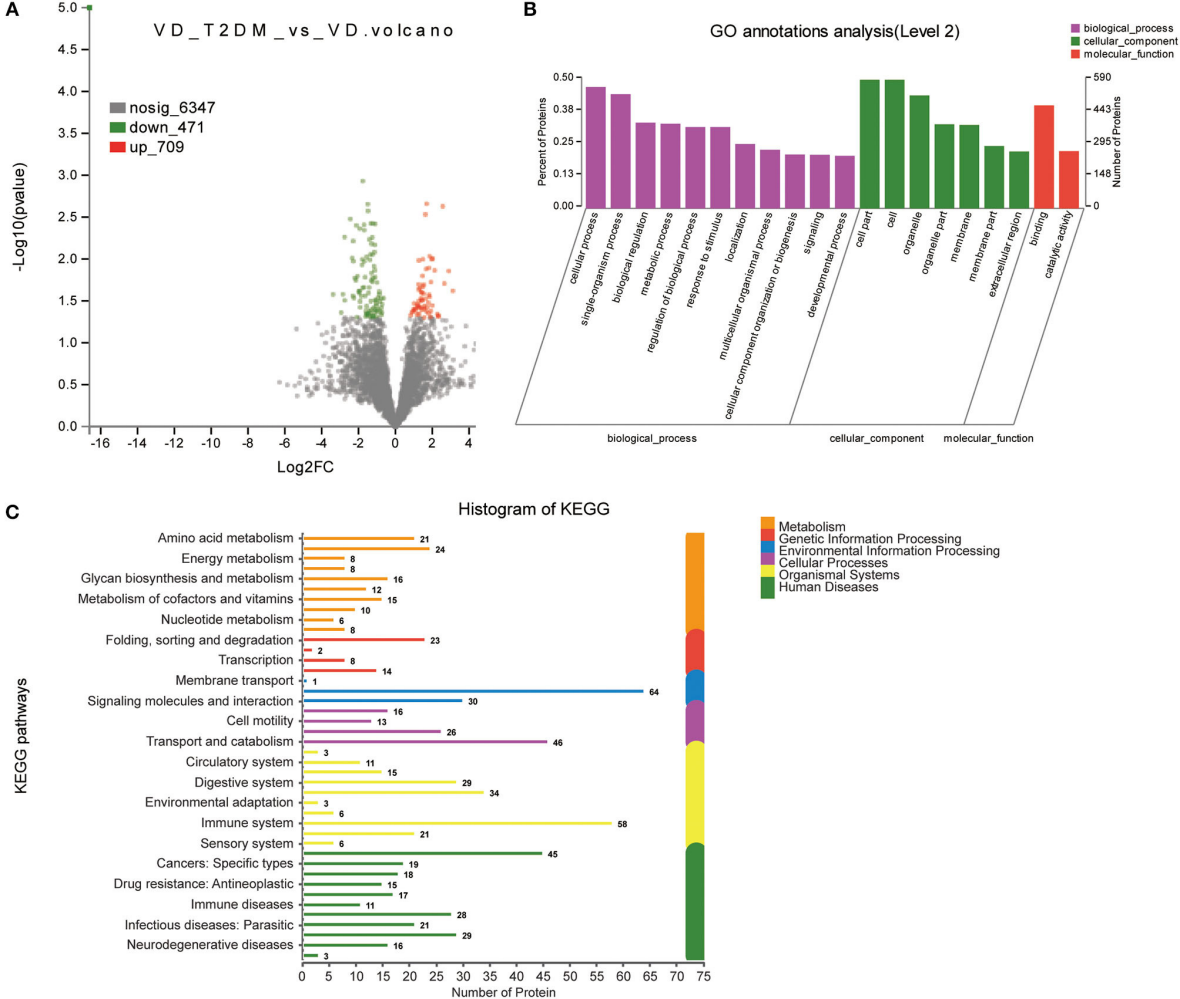
Volcano plots of quantified proteins and functional analysis of DEPs in the urine between subjects with VD+T2DM and VD (notes same as Figures 1–3).

In total, 1,180 proteins including 471 upregulated proteins and 709 downregulated proteins were differentially expressed in the VD+T2DM vs. VD groups (Figure 5A). The GO analysis using Blast2GO showed that terms related to the biological process were significantly enriched in the cellular process, the single-organism process, biological regulation, the metabolic process as similar as possible to two prior comparisons (Figure 5B). The canonical pathway analysis feature of KEGG showed that the signal transduction and the immune system pathway was significantly induced in the VD+T2DM group compared to VD, and 16 enriched proteins may participate in the neurodegenerative diseases (Figure 5C).

#### Common Differential Proteins of VD+T2DM Compared With NC, T2DM, and VD Groups Separately

**Figure 6:**
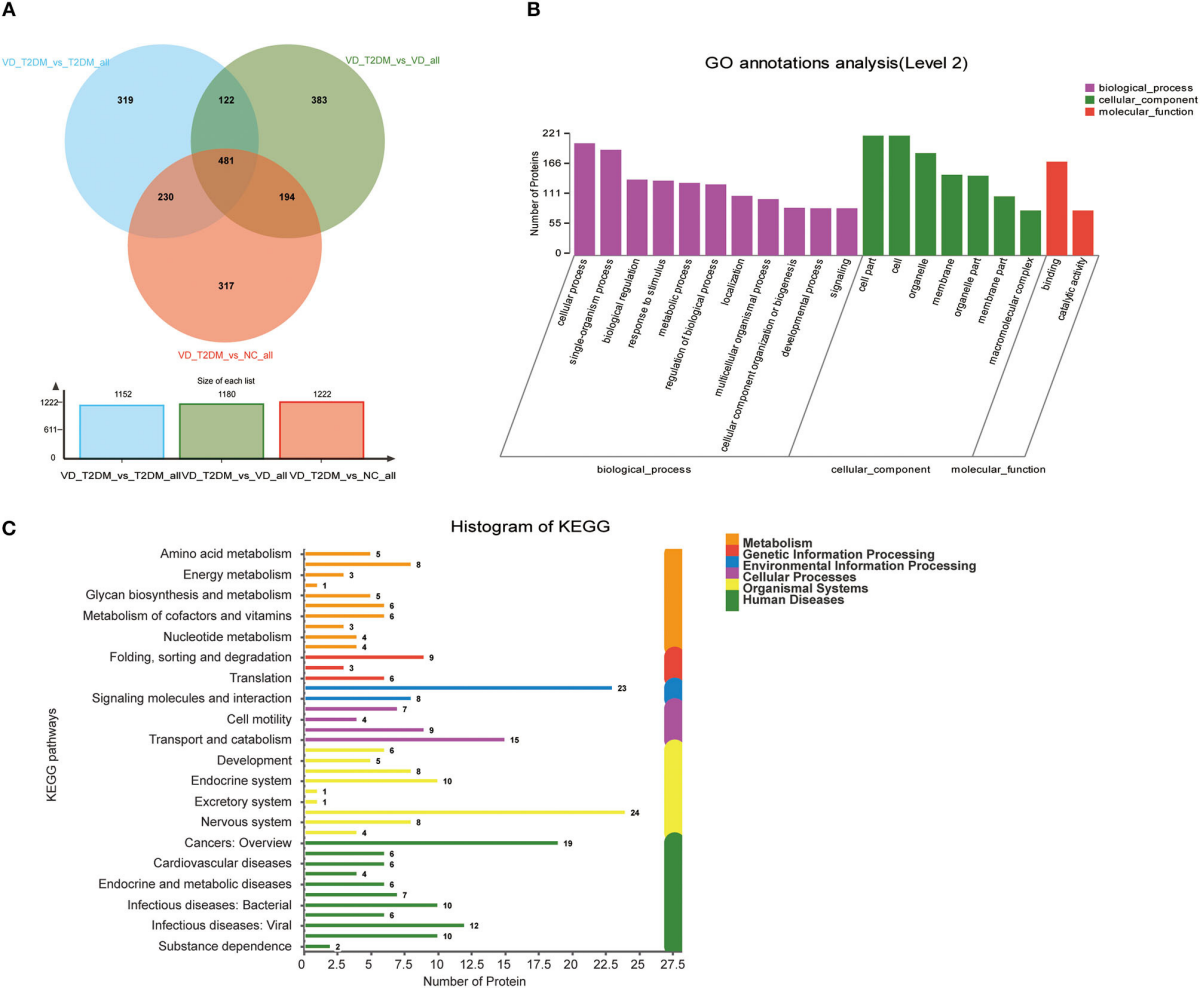
Volcano plots of quantified proteins and functional analysis of DEPs in urine between the VD+T2DM group and the other three groups. **(A)** Venn chart of urine-based proteome analysis identified from the VD+T2DM group vs. the other three groups. **(B)** GO enrichment analysis of differential urine-excretory proteins. Purple bars denote the enriched biological processes, green bars denote enriched cellular components, and red bars denote enriched molecular functions. Protein numbers enriched in each category are presented with the bars. **(C)** The DEPs are associated with diseases and signal transduction pathways. The number of proteins associated with each category is listed.

**Figure 7:**
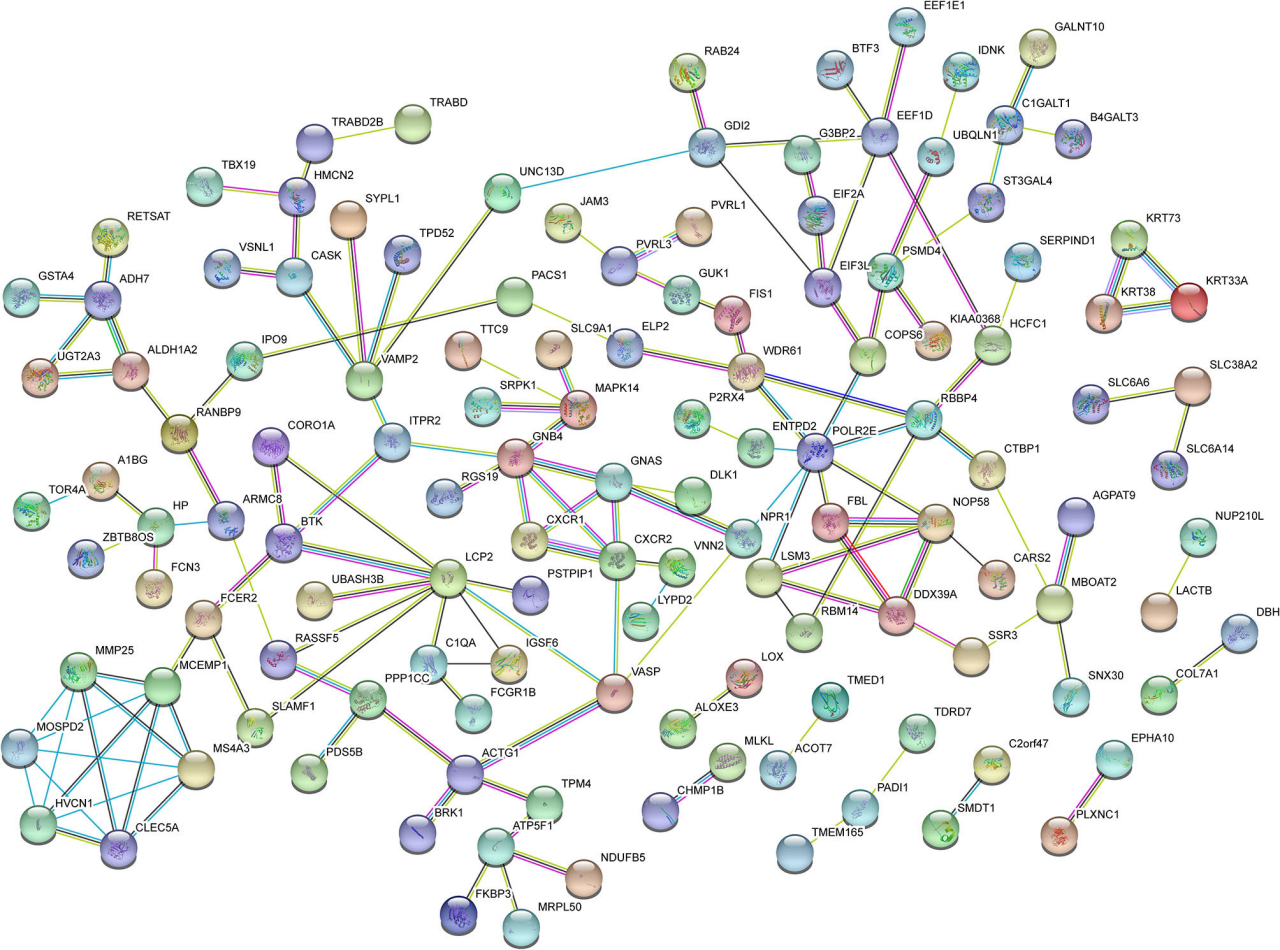
Protein–protein interaction (PPI) network analysis of DEPs in the urine between the VD+T2DM group and the other three groups using STRING. Each node represents a protein, and each edge represents a protein–protein association.

According to the comparison of the NC, T2DM, and VD groups, the DEPs of the DVD group with the other three groups were found, respectively, including 481 overlapped proteins as potential candidate biomarkers of DVD, which suggested that they may share some common molecular mechanisms contributing to the target disease (Figure 6A). The top 11 biological processes are similar to the above except the developmental process (Figure 6B). Twenty-four DEPs enriched in the immune system, 10 DEPs enriched in the endocrine system, eight DEPs enriched in the nervous system, as well as 23 DEPs enriched in signal transduction, 15 DEPs enriched in transport and catabolism, and 10 DEPs enriched in neurodegenerative diseases, were shown in Figure 6C. We used the STRING database that can generate a PPI network with tightly connected clusters shown in Figure 7. PVRL3-PVRL1, SERPINA1-TF, KRT73-KRT72, TF-SERPIND1, SERPINA1-SERPIND1, KRT72-KRT38, KRT73-KRT38, C7-C6, and MUC4-GALNT10 are the top nine network nodes based on a combined score (Figure 7 and Supplementary Table 4).

### Candidate Markers and Composite Biomarkers

**Figure 8:**
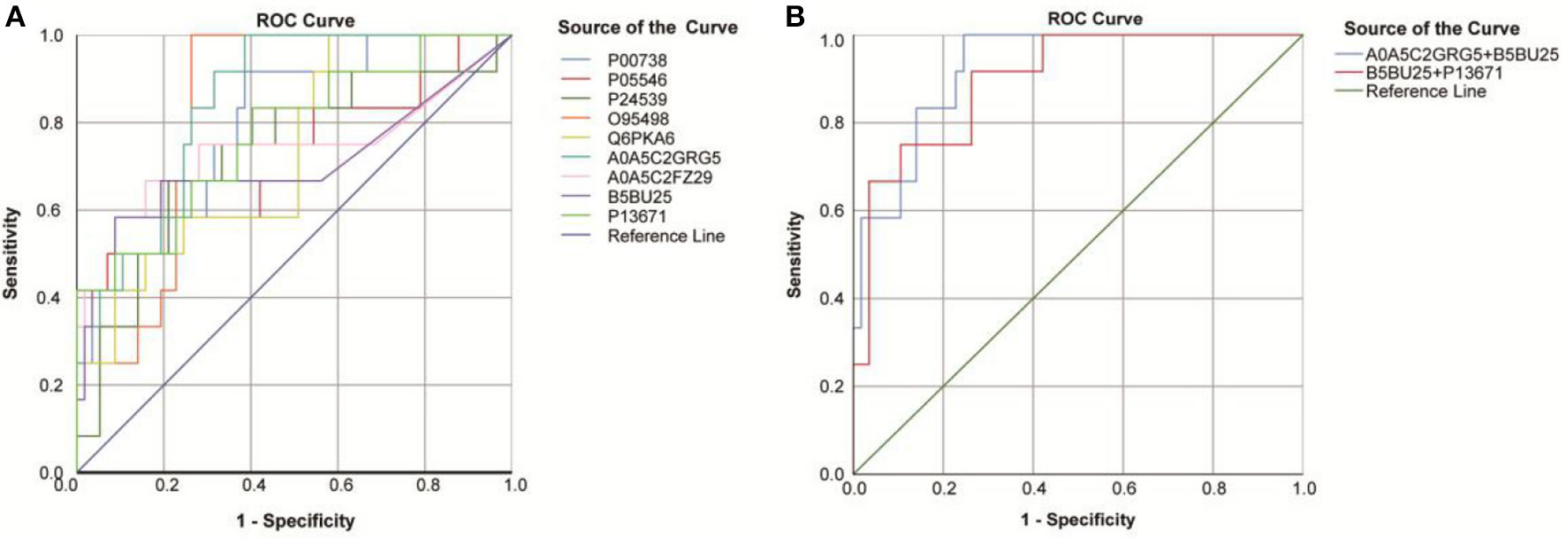
Receiver operating characteristic (ROC) curves obtained from each DEP and two top combining proteins, for the classification status of the DVD group from other groups. **(A)** ROC curves obtained from nine DEPs identified by the proteomic analysis. **(B)** ROC curves obtained from the two top combining proteins [A0A5C2GRG5 (no name)+U2AF2 and U2AF2+C6].

Fourteen DEPs were selected as potential candidate markers from 481 common DEPs, which were expressed in all groups (<50% of the samples in each group had a protein expression of zero) (Table 2). It included 10 upregulated and 4 downregulated proteins, among which the upregulated protein, Q969Q5, and all downregulated proteins were evaluated by the ROC curve with no statistical significance. Results on the performance of both a single marker and a CM (including 9 upregulated DEPs and 36 CMs) evaluated by the ROC curves were shown graphically in Figure 8A and Table 3, where the joint behaviors of standardized markers among disease DVD and other three groups were displayed. As clearly shown in Figure 8B and Table 3, the ability of two top CMs (A0A5C2GRG5+B5BU25 and B5BU25+P13671) to distinguish DVD from other diseases or NCs was better when two dimensions were used instead of one. Besides, the sensitivity of CM in all specificity ranges was improved compared with a single marker in the two ROC curves. In brief, nine upregulated DEPs (seven named in the GO database listed in Supplementary Table 6) as candidate biomarkers were identified in this study, which can also be combined as a CM to enhance their identification ability.

**Table 2:** Urinary DEPs between patients with VD+DM, patients with VD, patients with DM, and NCs.

**Accession**	**Description**	**VD_T2DM/NC**				**VD_T2DM/T2DM**				**VD_T2DM/VD**			
		**FC**	**Log2FC**	***P*_value**	**Regulate**	**FC**	**Log2FC**	***P*_value**	**Regulate**	**FC**	**Log2FC**	***P*_value**	**Regulate**
P00738	Haptoglobin OS=Homo sapiens OX = 9606 GN=HP PE = 1 SV = 1	3.625	1.858	0.02605	Up	4.222	2.078	0.01705	Up	6.336	2.664	0.01966	Up
P05546	Heparin cofactor 2 OS=Homo sapiens OX = 9606 GN=SERPIND1 PE = 1 SV = 3	2.631	1.396	0.009317	Up	3.932	1.975	0.001246	Up	2.893	1.533	0.02607	Up
P24539	ATP synthase F(0) complex subunit B1, mitochondrial OS=Homo sapiens OX = 9606 GN=ATP5PB PE = 1 SV = 2	1.976	0.9826	0.01767	Up	1.908	0.9321	0.02846	Up	1.988	0.9913	0.03989	Up
O95498	Vascular non-inflammatory molecule 2 OS=Homo sapiens OX = 9606 GN=VNN2 PE = 1 SV = 3	2.596	1.376	0.02664	Up	2.92	1.546	0.008341	Up	3.935	1.976	0.01007	Up
Q6PKA6	ALDH3A1 protein (fragment) OS=Homo sapiens OX = 9606 GN=ALDH3A1 PE = 2 SV = 1	2.642	1.402	0.02525	Up	2.456	1.296	0.04752	Up	3.104	1.634	0.02991	Up
A0A5C2GRG5	IG c376_light_IGLV3-21_IGLJ2 (fragment) OS=Homo sapiens OX = 9606 PE = 2 SV = 1	5.786	2.533	0.001428	Up	2.772	1.471	0.009193	Up	3.633	1.861	0.02833	Up
A0A5C2FZ29	IGL c2162_light_IGKV1-39_IGKJ1 (fragment) OS=Homo sapiens OX = 9606 PE = 2 SV = 1	6.385	2.675	0.000015	Up	3.231	1.692	0.00204	Up	2.823	1.497	0.01009	Up
B5BU25	U2 snRNP auxiliary factor large subunit OS=Homo sapiens OX = 9606 GN=U2AF2 PE = 2 SV = 1	5.159	2.367	0.01441	Up	5.778	2.531	0.008261	Up	7.47	2.901	0.01395	Up
Q969Q5	Ras-related protein Rab-24 OS=Homo sapiens OX = 9606 GN=RAB24 PE = 1 SV = 1	2.972	1.571	0.02592	Up	3.202	1.679	0.03026	Up	3.752	1.908	0.04909	Up
P13671	Complement component C6 OS=Homo sapiens OX = 9606 GN=C6 PE = 1 SV = 3	3.067	1.617	0.002368	Up	3.884	1.958	0.000715	Up	2.376	1.249	0.03629	Up
P07911	Uromodulin OS=Homo sapiens OX = 9606 GN=UMOD PE = 1 SV = 1	0.3283	−1.607	0.03101	Down	0.3213	−1.638	0.007548	Down	0.357	−1.486	0.002664	Down
A5PLM9	Cathepsin L1 OS=Homo sapiens OX = 9606 GN=CTSL1 PE = 2 SV = 1	0.2151	−2.217	0.01521	Down	0.167	−2.582	0.04136	Down	0.1838	−2.444	0.003328	Down
B3KY78	cDNA FLJ46304 fis, clone TESTI4037949, highly similar to Kelch-like protein 8 OS=Homo sapiens OX = 9606 PE = 2 SV = 1	0.3246	−1.623	0.03735	Down	0.3582	−1.481	0.03917	Down	0.3082	−1.698	0.003907	Down
A0A384MDZ8	Epididymis secretory sperm binding protein OS=Homo sapiens OX = 9606 PE = 2 SV = 1	0.2607	−1.94	0.008273	Down	0.2917	−1.777	0.04555	Down	0.3534	−1.501	0.002213	Down

**Table 3:** AUC statistics per protein and combined proteins, for the classification status of DVD group from other groups.

**Variable protein name/gene name**	**Optimal cutpoint**	**Sensitivity**	**1-Specificity**	**Euclidean's index**	**AUC**	**CI. low**	**CI. high**	***P*-value**
P00738	885,561,904	0.917	0.386	0.155885	0.785	0.656	0.915	0.002
P05546	1268692,696	0.583	0.088	0.181633	0.725	0.537	0.913	0.015
P24539	1,251,574,887	0.667	0.211	0.15541	0.730	0.561	0.898	0.013
O95498	389491398.9	1	0.263	0.069169	0.829	0.731	0.927	0.000
Q6PKA6	452319949.4	0.583	0.246	0.234405	0.731	0.582	0.880	0.012
A0A5C2GRG5	170,596,212	0.833	0.263	0.097058	0.848	0.750	0.946	0.000
A0A5C2FZ29	186896857.4	0.667	0.158	0.135853	0.737	0.537	0.937	0.010
B5BU25	28141947.62	0.667	0.193	0.148138	0.703	0.498	0.908	0.028
P13671	74615703.49	0.667	0.263	0.180058	0.773	0.620	0.927	0.003
P00738+P05546	0.1391723	0.75	0.175	0.093125	0.779	0.599	0.959	0.002
P00738+P24539	0.1041441	0.917	0.298	0.095693	0.858	0.748	0.968	0.000
P00738+O95498	0.1008949	0.917	0.316	0.106745	0.857	0.758	0.955	0.000
P00738+Q6PKA6	0.1210192	0.75	0.211	0.107021	0.842	0.736	0.948	0.000
P00738+A0A5C2GRG5	0.1729689	0.667	0.123	0.126018	0.833	0.716	0.951	0.000
P00738+A0A5C2FZ29	0.2170247	0.667	0.14	0.130489	0.765	0.586	0.943	0.004
P00738+B5BU25	0.1094936	0.833	0.193	0.065138	**0.886**	0.795	0.977	0.000
P00738+P13671	0.1254628	0.75	0.281	0.141461	0.781	0.623	0.938	0.002
P05546+P24539	0.1269705	0.75	0.316	0.162356	0.773	0.623	0.924	0.003
P05546+O95498	0.248127	0.583	0.088	0.181633	0.781	0.632	0.930	0.002
P05546+Q6PKA6	0.1240923	0.75	0.263	0.131669	0.794	0.644	0.943	0.001
P05546+A0A5C2GRG5	0.1329428	0.667	0.211	0.15541	0.768	0.605	0.930	0.004
P05546+A0A5C2FZ29	0.1077113	0.833	0.211	0.07241	0.819	0.638	1.000	0.001
P05546+B5BU25	0.3128123	0.833	0.053	0.030698	0.925	0.838	1.000	0.000
P05546+P13671	0.1352016	0.75	0.263	0.131669	0.791	0.630	0.952	0.002
P24539+O95498	0.1303219	0.917	0.281	0.08585	0.819	0.695	0.943	0.001
P24539+Q6PKA6	0.1686308	0.667	0.246	0.171405	0.740	0.566	0.913	0.009
P24539+A0A5C2GRG5	0.1009088	0.917	0.316	0.106745	0.842	0.729	0.955	0.000
P24539+A0A5C2FZ29	0.1652986	0.75	0.193	0.099749	**0.887**	0.790	0.985	0.000
P24539+B5BU25	0.1958116	0.833	0.14	0.047489	**0.887**	0.792	0.983	0.000
P24539+P13671	0.1278419	0.667	0.333	0.221778	0.792	0.657	0.927	0.002
O95498+Q6PKA6	0.1110988	0.917	0.316	0.106745	0.836	0.735	0.937	0.000
O95498+A0A5C2GRG5	0.0934431	0.833	0.316	0.127745	0.846	0.744	0.949	0.000
O95498+A0A5C2FZ29	0.1131648	0.75	0.211	0.107021	0.825	0.686	0.963	0.000
O95498+B5BU25	0.102268	0.917	0.281	0.08585	0.883	0.792	0.974	0.000
O95498+P13671	0.1913238	0.667	0.158	0.135853	0.836	0.708	0.965	0.000
Q6PKA6+A0A5C2GRG5	0.1053598	0.833	0.228	0.079873	0.874	0.780	0.969	0.000
Q6PKA6+A0A5C2FZ29	0.1107437	0.75	0.211	0.107021	0.873	0.762	0.984	0.000
Q6PKA6+B5BU25	0.1230676	0.833	0.14	0.047489	0.864	0.742	0.986	0.000
Q6PKA6+P13671	0.1606209	0.667	0.211	0.15541	0.851	0.735	0.967	0.000
A0A5C2GRG5+A0A5C2FZ29	0.1775901	0.75	0.123	0.077629	0.825	0.676	0.973	0.000
A0A5C2GRG5+B5BU25	0.140421	0.833	0.14	0.047489	**0.924**	0.858	0.990	0.000
A0A5C2GRG5+P13671	0.2018078	0.667	0.105	0.121914	0.816	0.673	0.959	0.001
A0A5C2FZ29+B5BU25	0.1353738	0.75	0.175	0.093125	0.882	0.795	0.968	0.000
A0A5C2FZ29+P13671	0.2593937	0.667	0.088	0.118633	0.788	0.606	0.970	0.002
B5BU25+P13671	0.1484973	0.75	0.105	0.073525	**0.898**	0.810	0.985	0.000

*AUC, area under the curve; CI, 95% confidence interval. Bold represents the top 5 AUC*.

## Discussion

Type 2 diabetes mellitus is a risk factor for both VD and AD, although prior studies of AD pathologic markers have not provided sufficient evidence (Bellou et al., 2017). In addition to superposition or synergistic interaction with various factors, such as old age, hypertension, and total cholesterol, T2DM affects the cognitive function of patients with dementia, and its comorbidities can also aggravate the clinical manifestations of patients with VD or AD (Biessels and Despa, 2018). Therefore, identifying available early diagnostic biomarkers would provide an effective opportunity for the therapeutic intervention of DVD and update our understanding of how T2DM is associated with dementia and the underlying pathophysiological mechanisms.

In this study, of all 4,744 urinary proteins successfully detected, 481 differential urinary protein biomarkers of DVD compared with the other three groups were predicted based on the HPLC-MS/MS analysis of proteomic data of DVD. The GO enrichment analysis of these proteins showed that they were significantly enriched in the cellular process, the single-organism process, biological regulation, the metabolic process, the regulation of the biological process, response to the stimulus, localization, cellular component organization or biogenesis, and signaling. The KEGG pathway analysis revealed that they are associated with amino acid metabolism; energy metabolism; the biosynthesis of glycans and metabolism; the metabolism of cofactors and vitamins; nucleotide metabolism folding, sorting, and degradation; translation; signaling molecules and interaction; cell motility; transportation and catabolism; development; the endocrine system; the excretory system; and the nervous system and are relevant to various diseases, such as cancers, cardiovascular diseases (CVDs), endocrine and metabolic diseases, infectious diseases, such as bacterial infectious diseases, and neurodegenerative diseases, suggesting that some of the candidates in this study were involved in multiple disease processes and may have limited disease specificity. In addition, PVRL3-PVRL1, SERPINA1-TF, KRT73-KRT72, TF-SERPIND1, SERPINA1-SERPIND1, KRT72-KRT38, KRT73-KRT38, C7-C6, and MUC4-GALNT10 are the top nine network nodes in the PPI network analysis. Finally, out of these proteins, nine potential proteins (including HP, SERPIND, ATP5PB, VNN2, ALDH3A1, U2AF2, C6, A0A5C2GRG5 (no name), and A0A5C2FZ29 (no name) and two CMs were speculated to be potential urinary biomarkers of DVD and were chosen from the urine of DVD patients vs. other three controls by quantification of the ROC curve.

Haptoglobin (HP) is an acute hemoglobin-binding protein produced by the liver that exhibits anti-inflammatory activities and plays a crucial role in protecting against heme-driven oxidative stress (Sadrzadeh and Bozorgmehr, 2004; Arredouani et al., 2005), which is involved in the development of diabetic vascular complications (Levy et al., 2000). HP has two common alleles, HP1 allele and HP2 allele, which have different antioxidant capacities, and three phenotypes, namely HP 1-1, HP 2-1, and HP 2-2 (Sadrzadeh and Bozorgmehr, 2004). A key difference between the two alleles is that the protein product produced by the HP1 allele has a stronger antioxidant ability than that produced by the HP2 allele (Melamed-Frank et al., 2001). As early as 1973, it was reported that both HP 1-2 and HP 2-2 genotypes were significantly increased in patients with early-onset dementia (Op den Velde and Stam, 1973). Similar results were obtained in diabetic patients by many following studies in recent years, and HP 1-1 genotype was considered to be associated with poorer cognitive functioning due to greater susceptibility to cerebrovascular diseases, whereas the HP2 allele enhanced the gene expression of an angiogenic factor in endothelial progenitor cells and may improve blood perfusion and recovery after an ischemic injury (German et al., 2007; Ravona-Springer et al., 2013; Wang et al., 2015; Beeri et al., 2018). In addition, it was suggested that the progressive increase of HP serum levels could be related to the progression of neurodegenerative diseases in a recent case-control study (Zhu et al., 2018). It may have the ability to inhibit the formation of amyloid fibrils and protect nerve cells from Aβ-induced toxicity by forming stable high-molecular weight complexes with misfolded proteins when this system of defense is overwhelmed under a pathologic circumstance (Yerbury et al., 2009). Furthermore, many previous studies have suggested that HP phenotypes may be significant independent risk factors for CVDs in individuals with DM, and the incidence of CVD in individuals with HP 2-2 phenotype was significantly higher than that in individuals with HP 2-1 or HP 1-1 phenotypes (Levy et al., 2002; Cahill et al., 2013; Costacou and Howard, 2020). In this study, elevated urinary levels of subjects with DM were consistent with the above data, but the genotyping of these individuals needs to be further clarified to understand the different functional mechanisms of HP.

Heparin Cofactor II (HCII, designated as SERPIND1 in humans) is another key upregulated protein identified in this study. It is a serine protease inhibitor (serpin), first detected in 1974 (Briginshaw and Shanberge, 1974), that mainly produced in the liver and secreted into the bloodstream and that has structural similarities to antithrombin (Tollefsen, 2007). HC has been demonstrated to play a vascular protective role against vascular remodeling and atherosclerosis, which can form a bimolecular complex with dermatan sulfate (DS) inhibiting the action of thrombin (Tollefsen, 2007). However, HCII is inactive against other proteases involved in coagulation or fibrinolysis (Parker and Tollefsen, 1985). Since DS is mainly generated by smooth muscle cells and fibroblasts, finally depositing in the matrix of vascular intima and media, HCII could counteract the effect of thrombin when the walls of blood vessels are damaged (Aihara et al., 2004). Therefore, it is speculated in this study whether the elevation of this marker suggests an increased protective stress after vascular injury and predicts a worse cognitive impairment outcome in patients with DVD. There are still various issues of HCII which remains to be elucidated, including the identification of major cell and tissue targets, as well as physiological conditions (such as hypofibrinolysis and inflammation) involved in the reaction of HCII in the subjects with DVD.

Mitochondrial abnormalities have been reported to be a causative factor for diabetes, stroke, and cognitive deterioration (De Felice and Ferreira, 2014; Yang et al., 2018). Mitochondria are well-known dynamic organelles with a variety of functions, most notably ATP production. ATP5F1 (ATP5PB) is an ATP synthase subunit b which represents the fifth complex (F0) of the mitochondrial electron transport chain. ATP synthase participates in the oxidative phosphorylation of ADP into ATP in the inner mitochondrial membrane of cells, which plays a central role in the supply of ATP for the maintenance of brain function (Chow et al., 2017). ATP5F1 is annotated in the KEGG database to be associated with a variety of neurodegenerative diseases, such as AD, Parkinson's disease, and Huntington's disease, and is involved in the pathway of oxidative phosphorylation, metabolic pathways, and the pathway of thermogenesis (hsa: 515). However, there is still limited reported about how ATP5F1 acts in the pathological state and whether its elevated levels in our study reveal mitochondrial dysfunction contributing to DVD compared with other groups.

Numerous functional studies demonstrate a role for Vanin genes [Vanin-1 (VNN1), Vanin-2 (VNN2), Vanin-3 (VNN3)] in inflammation, oxidative stress, cell migration, and various diseases such as diabetes and CVDs (Kaskow et al., 2012). VNN2, belying its original description as a vascular non-inflammatory molecule, is thought to participate in inflammation and leukocyte migration, and its mRNA expression has been verified in almost all tissues, particularly neutrophils (Suzuki et al., 1999). Gpi80, the product of VNN2, showed aggregation on the surface of activated neutrophils undergoing migration, which may elevate the level of β2 integrin and regulate the adhesion and migration of neutrophils (Kaskow et al., 2012). Since chronic inflammation is considered to be one of the main pathological mechanisms of diabetes and dementia, higher expression of VNN2 may be related to pathological events.

ALDH3A1 is a member of the aldehyde dehydrogenase (ALDH) superfamily, which plays a crucial role in the metabolism of diverse endogenous and exogenous aldehydes (Koppaka et al., 2012). With the capacity of medium-chain aliphatic and aromatic aldehyde metabolizing enzyme, ALDH3A1 is abundant in the upper respiratory tract, cornea, digestive tract, esophagus, and stomach and is reported to be highly expressed in non-small cell lung cancer (NSCLC) as a prognostic marker for patients with NSCLC (Pappa et al., 2003; Rebollido-Rios et al., 2020). Most studies on this gene have focused on its role in increasing the resistance of tumor cells to anticancer drugs (Koppaka et al., 2012), assisting ALDH2 in acetaldehyde and ethanol metabolism (Chen et al., 2015), and its high expression representing a poor clinical prognosis of cancer (Rebollido-Rios et al., 2020), as well as its function in protecting intraocular tissues from exposure to UV radiation and reactive oxygen species-induced damage (Chen et al., 2013). However, the role of ALDH3A1 in neurodegenerative diseases has not been reported.

U2AF2 (also known as U2AF65), the RNA-binding protein (RBP), is essential for splicing decisions, as it can recognize 3' splice sites and recruit the spliceosome (Sutandy et al., 2018). Cancer mutations found in U2AF2 have been mapped to structural changes in RNA recognition motifs (RRMs) affecting the selection at the 30 splicing site (30SS) (Glasser et al., 2017). Similarly, the function of U2AF2 in neurological diseases has not been reported in the literature.

As one of the terminal complement components, C6 combines with C5b and then binds to C7, C8, and C9 to form the membrane attack complex (MAC), which participates in the innate immune response, promotes the inflammatory response, and coordinates the defense against pathogens (Moya-Quiles et al., 2013). However, unrestrained immune activation may cause a harmful chronic inflammatory environment that results in upregulated expression of various complement proteins in neurodegeneration such as AD and Huntington's disease (Gasque et al., 2000).

Nonetheless, several limitations of the study are considered. First, clinically, since AD, VD, and mixed dementia (MD) may have overlapping pathological mechanisms and diagnostic markers, it is very difficult to make a definite diagnosis. When patients with VD were enrolled, it was troublesome to exclude those with MD, and the molecular mechanisms studied may be confusing. Second, the length and different stages of the disease may affect the severity of VD in patients with diabetes, which means that the biomarkers in urine are likely to be different. No in-depth stratification was conducted according to the history of DM and the type, stage, and location of VD or comorbidities, such as hypertension, heart disease, and other confounding factors in the current study. Third, though nine DVD-related urinary proteins were detected and predicted by the LC-MS/MS method and quantified through ROC curves, they were not strongly validated to be differentially expressed in urine samples of DVD by experiments such as WB, qPCR, and ELISA. It is necessary to conduct follow-up studies on samples using other skills to validate the pathology specificity of a single protein. Besides, variations of 13 urinary proteins were calculated and represented with intrapersonal CVs from each dataset in a box-plot format (Supplementary Figure 1). Proteins with relatively large CV values in the group may indicate that these proteins have a poor homogeneity in the same group and may affect the reliability of statistical results. A central issue to be addressed in urinary proteomics is the proteomic variability, which has been illustrated in many previous studies (Nagaraj and Mann, 2011). Determination of variability is due to technical variation or many physiological factors, such as daily, gender, aging, diet, exercise, and intra- and inter-individual variations (Nagaraj and Mann, 2011; Oeyen et al., 2019; Shao et al., 2019), which need to be reduced by expanding the sample size, stratified analysis, normalized sampling procedure, and more stringent values of *p* in our future studies.

## Conclusion

To conclude, our work demonstrated an effective and specific way to discover urinary upregulated biomarkers of DVD compared to subjects with VD, subjects with T2DM, and NCs, which may contribute to the early diagnosis and intervention of this disease. We have successfully used the HPLC-MS/MS analysis approach to confidently identify 4,744 proteins and subsequently identified 481 DEPs to distinguish from the other three groups. Besides, we were able to obtain the statistically significant difference of nine candidate proteins [including HP, SERPIND, ATP5PB, VNN2, ALDH3A1, U2AF2, C6, A0A5C2GRG5 (no name), and A0A5C2FZ29 (no name)] and two CMs (A0A5C2GRG5+U2AF2 and U2AF2+C6) between DVD and other controls based on the analysis of ROC curves. Our results documented the upregulation of seemingly protective and deleterious candidate markers simultaneously in the urinary samples of subjects with DVD, identifying the key molecular substrates for different pathological processes. Although these preliminary findings must be validated in a much larger and diverse patient population using multiple methods, they suggest that a range of proteins can be generated and developed into specific biomarkers that could ultimately help in clinical diagnosis and monitoring of the progression of DVD.

## Data Availability Statement

The original contributions presented in the study are publicly available. This data can be found at: PXD022189.

## Ethics Statement

The studies involving human participants were reviewed and approved by Ethics Committee of Second Xiangya Hospital, Central South University. The patients/participants provided their written informed consent to participate in this study.

## Author Contributions

YZ designed the research and determined the structure of the paper. RC selected the references and contributed to the writing. YY collected the clinical data. WX and BZ helped to analyze the results of the experiment. YS and LZ contributed to the revision and finalization of the article. All authors contributed to the article and approved the submitted version.

## Conflict of Interest

The authors declare that the research was conducted in the absence of any commercial or financial relationships that could be construed as a potential conflict of interest.

## References

[B1] Abdi F. Quinn J. F. Jankovic J. McIntosh M. Leverenz J. B. Peskind E.. (2006). Detection of biomarkers with a multiplex quantitative proteomic platform in cerebrospinal fluid of patients with neurodegenerative disorders. J. Alzheimers Dis. 9, 293–348. 10.3233/JAD-2006-9309 16914840

[B2] Ahtiluoto S. Polvikoski T. Peltonen M. Solomon A. Tuomilehto J. Winblad B.. (2010). Diabetes, Alzheimer disease, and vascular dementia: a population-based neuropathologic study. Neurology 75, 1195–1202. 10.1212/WNL.0b013e3181f4d7f8 20739645

[B3] Aihara K. Azuma H. Takamori N. Kanagawa Y. Akaike M. Fujimura M.. (2004). Heparin cofactor II is a novel protective factor against carotid atherosclerosis in elderly individuals. Circulation 109, 2761–2765. 10.1161/01.CIR.0000129968.46095.F3 15148272

[B4] An M. Gao Y. (2015). Urinary biomarkers of brain diseases. Genomics Proteomics Bioinformatics 13, 345–354. 10.1016/j.gpb.2015.08.005 26751805 PMC4747650

[B5] Arredouani M. S. Kasran A. Vanoirbeek J. A. Berger F. G. Baumann H. Ceuppens J. L. (2005). Haptoglobin dampens endotoxin-induced inflammatory effects both *in vitro* and *in vivo*. Immunology 114, 263–271. 10.1111/j.1365-2567.2004.02071.x 15667571 PMC1782073

[B6] Beeri M. S. Lin H. M. Sano M. Ravona-Springer R. Liu X. Bendlin B. B.. (2018). Association of the haptoglobin gene polymorphism with cognitive function and decline in elderly African American adults with type 2 diabetes: findings from the action to control cardiovascular risk in diabetes-memory in diabetes (ACCORD-MIND) study. JAMA Netw. Open 1:e184458. 10.1001/jamanetworkopen.2018.4458 30646354 PMC6324406

[B7] Bellou V. Belbasis L. Tzoulaki I. Middleton L. T. Ioannidis J. P. A. Evangelou E. (2017). Systematic evaluation of the associations between environmental risk factors and dementia: an umbrella review of systematic reviews and meta-analyses. Alzheimers Dement 13, 406–418. 10.1016/j.jalz.2016.07.152 27599208

[B8] Biessels G. J. Despa F. (2018). Cognitive decline and dementia in diabetes mellitus: mechanisms and clinical implications. Nat. Rev. Endocrinol. 14, 591–604. 10.1038/s41574-018-0048-7 30022099 PMC6397437

[B9] Biessels G. J. Strachan M. W. Visseren F. L. Kappelle L. J. Whitmer R. A. (2014). Dementia and cognitive decline in type 2 diabetes and prediabetic stages: towards targeted interventions. Lancet Diabetes Endocrinol. 2, 246–255. 10.1016/S2213-8587(13)70088-3 24622755

[B10] Boulton A. J. (1998). Guidelines for diagnosis and outpatient management of diabetic peripheral neuropathy. European Association for the study of diabetes. Neurodiab. Diabetes Metab. 24(Suppl. 3), 55–65. 9881234

[B11] Briginshaw G. F. Shanberge J. N. (1974). Identification of two distinct heparin cofactors in human plasma. Separation and partial purification. Arch. Biochem. Biophys. 161, 683–690. 10.1016/0003-9861(74)90354-3 4839055

[B12] Cahill L. E. Levy A. P. Chiuve S. E. Jensen M. K. Wang H. Shara N. M.. (2013). Haptoglobin genotype is a consistent marker of coronary heart disease risk among individuals with elevated glycosylated hemoglobin. J. Am. Coll. Cardiol. 61, 728–737. 10.1016/j.jacc.2012.09.063 23312704 PMC3678553

[B13] Capello M. Bantis L. E. Scelo G. Zhao Y. Li P. Dhillon D. S.. (2017). Sequential validation of blood-based protein biomarker candidates for early-stage pancreatic cancer. J. Natl. Cancer Inst. 109:djw266. 10.1093/jnci/djw266 28376157 PMC5441297

[B14] Chen C. H. Cruz L. A. Mochly-Rosen D. (2015). Pharmacological recruitment of aldehyde dehydrogenase 3A1 (ALDH3A1) to assist ALDH2 in acetaldehyde and ethanol metabolism *in vivo*. Proc. Natl. Acad. Sci. U.S.A. 112, 3074–3079. 10.1073/pnas.1414657112 25713355 PMC4364197

[B15] Chen Y. Thompson D. C. Koppaka V. Jester J. V. Vasiliou V. (2013). Ocular aldehyde dehydrogenases: protection against ultraviolet damage and maintenance of transparency for vision. Prog. Retin. Eye Res. 33, 28–39. 10.1016/j.preteyeres.2012.10.001 23098688 PMC3570594

[B16] Cheng G. Huang C. Deng H. Wang H. (2012). Diabetes as a risk factor for dementia and mild cognitive impairment: a meta-analysis of longitudinal studies. Intern. Med. J. 42, 484–491. 10.1111/j.1445-5994.2012.02758.x 22372522

[B17] Chow J. Rahman J. Achermann J. C. Dattani M. T. Rahman S. (2017). Mitochondrial disease and endocrine dysfunction. Nat. Rev. Endocrinol. 13, 92–104. 10.1038/nrendo.2016.151 27716753

[B18] Costacou T. Howard B. V. (2020). Should the haptoglobin genotype be considered in setting glycemic goals for diabetes patients? J. Am. Coll. Cardiol. 75, 522–524. 10.1016/j.jacc.2019.11.052 32029135

[B19] De Felice F. G. Ferreira S. T. (2014). Inflammation, defective insulin signaling, and mitochondrial dysfunction as common molecular denominators connecting type 2 diabetes to Alzheimer disease. Diabetes 63, 2262–2272. 10.2337/db13-1954 24931033

[B20] Eng J. K. McCormack A. L. Yates J. R. (1994). An approach to correlate tandem mass spectral data of peptides with amino acid sequences in a protein database. J. Am. Soc. Mass Spectrom 5, 976–989. 10.1016/1044-0305(94)80016-2 24226387

[B21] Gao Y. (2019). Now is the time to test early urinary biomarkers in large-scale human samples. Sci. China Life Sci. 62, 851–853. 10.1007/s11427-019-9562-y 31114936

[B22] Gasque P. Dean Y. D. McGreal E. P. VanBeek J. Morgan B. P. (2000). Complement components of the innate immune system in health and disease in the CNS. Immunopharmacology 49, 171–186. 10.1016/S0162-3109(00)80302-1 10904116

[B23] German D. C. Gurnani P. Nandi A. Garner H. R. Fisher W. Diaz-Arrastia R.. (2007). Serum biomarkers for Alzheimer's disease: proteomic discovery. Biomed. Pharmacother. 61, 383–389. 10.1016/j.biopha.2007.05.009 17614251

[B24] Glasser E. Agrawal A. A. Jenkins J. L. Kielkopf C. L. (2017). Cancer-associated mutations mapped on high-resolution structures of the U2AF2 RNA recognition motifs. Biochemistry 56, 4757–4761. 10.1021/acs.biochem.7b00551 28850223 PMC6005654

[B25] Hugo J. Ganguli M. (2014). Dementia and cognitive impairment: epidemiology, diagnosis, and treatment. Clin. Geriatr. Med. 30, 421–442. 10.1016/j.cger.2014.04.001 25037289 PMC4104432

[B26] Jensen L. J. Kuhn M. Stark M. Chaffron S. Creevey C. Muller J.. (2009). STRING 8–a global view on proteins and their functional interactions in 630 organisms. Nucleic Acids Res. 37, D412–416. 10.1093/nar/gkn760 18940858 PMC2686466

[B27] Kaskow B. J. Proffitt J. M. Blangero J. Moses E. K. Abraham L. J. (2012). Diverse biological activities of the vascular non-inflammatory molecules - the Vanin pantetheinases. Biochem. Biophys. Res. Commun. 417, 653–658. 10.1016/j.bbrc.2011.11.099 22155241 PMC3259148

[B28] Koppaka V. Thompson D. C. Chen Y. Ellermann M. Nicolaou K. C. Juvonen R. O.. (2012). Aldehyde dehydrogenase inhibitors: a comprehensive review of the pharmacology, mechanism of action, substrate specificity, and clinical application. Pharmacol. Rev. 64, 520–539. 10.1124/pr.111.005538 22544865 PMC3400832

[B29] Krey J. F. Wilmarth P. A. Shin J. B. Klimek J. Sherman N. E. Jeffery E. D.. (2014). Accurate label-free protein quantitation with high- and low-resolution mass spectrometers. J. Proteome Res. 13, 1034–1044. 10.1021/pr401017h 24295401 PMC3946283

[B30] Leng W. Ni X. Sun C. Lu T. Malovannaya A. Jung S. Y.. (2017). Proof-of-concept workflow for establishing reference intervals of human urine proteome for monitoring physiological and pathological changes. EBioMedicine 18, 300–310. 10.1016/j.ebiom.2017.03.028 28396014 PMC5405183

[B31] Levy A. P. Hochberg I. Jablonski K. Resnick H. E. Lee E. T. Best L.. (2002). Haptoglobin phenotype is an independent risk factor for cardiovascular disease in individuals with diabetes: the Strong Heart Study. J. Am. Coll. Cardiol. 40, 1984–1990. 10.1016/S0735-1097(02)02534-2 12475459

[B32] Levy A. P. Roguin A. Hochberg I. Herer P. Marsh S. Nakhoul F. M.. (2000). Haptoglobin phenotype and vascular complications in patients with diabetes. N. Engl. J. Med. 343, 969–970. 10.1056/NEJM200009283431313 11012324

[B33] Melamed-Frank M. Lache O. Enav B. I. Szafranek T. Levy N. S. Ricklis R. M.. (2001). Structure-function analysis of the antioxidant properties of haptoglobin. Blood 98, 3693–3698. 10.1182/blood.V98.13.3693 11739174

[B34] Moya-Quiles M. R. Bernardo-Pisa M. V. Martínez P. Gimeno L. Bosch A. Salgado G.. (2013). Complement component C6 deficiency in a Spanish family: implications for clinical and molecular diagnosis. Gene 521, 204–206. 10.1016/j.gene.2013.03.027 23537992

[B35] Nagaraj N. Mann M. (2011). Quantitative analysis of the intra- and inter-individual variability of the normal urinary proteome. J. Proteome Res. 10, 637–645. 10.1021/pr100835s 21126025

[B36] Ninomiya T. (2014). Diabetes mellitus and dementia. Curr. Diab. Rep. 14, 487. 10.1007/s11892-014-0487-z 24623199

[B37] O'Brien J. T. Thomas A. (2015). Vascular dementia. Lancet 386, 1698–1706. 10.1016/S0140-6736(15)00463-8 26595643

[B38] Oeyen E. Willems H. t Kindt R. Sandra K. Boonen K. Hoekx L.. (2019). Determination of variability due to biological and technical variation in urinary extracellular vesicles as a crucial step in biomarker discovery studies. J. Extracell. Vesicles 8:1676035. 10.1080/20013078.2019.1676035 31681468 PMC6807909

[B39] Op den Velde W. Stam F. C. (1973). Haptoglobin types and subtypes in Alzheimer's disease and senile dementia. Humangenetik 20, 25–30. 10.1007/BF00280872 4776526

[B40] Pappa A. Estey T. Manzer R. Brown D. Vasiliou V. (2003). Human aldehyde dehydrogenase 3A1 (ALDH3A1): biochemical characterization and immunohistochemical localization in the cornea. Biochem. J. 376(Pt 3), 615–623. 10.1042/bj20030810 12943535 PMC1223798

[B41] Parker K. A. Tollefsen D. M. (1985). The protease specificity of heparin cofactor II. Inhibition of thrombin generated during coagulation. J. Biol. Chem. 260, 3501–3505. 10.1016/S0021-9258(19)83650-5 3838315

[B42] Peters S. A. Huxley R. R. Woodward M. (2014). Diabetes as a risk factor for stroke in women compared with men: a systematic review and meta-analysis of 64 cohorts, including 775,385 individuals and 12,539 strokes. Lancet 383, 1973–1980. 10.1016/S0140-6736(14)60040-4 24613026

[B43] Pohjasvaara T. Mäntylä R. Ylikoski R. Kaste M. Erkinjuntti T. (2000). Comparison of different clinical criteria (DSM-III, ADDTC, ICD-10, NINDS-AIREN, DSM-IV) for the diagnosis of vascular dementia. National Institute of Neurological Disorders and Stroke-Association Internationale pour la Recherche et l'Enseignement en Neurosciences. Stroke 31, 2952–2957. 10.1161/01.STR.31.12.2952 11108755

[B44] Prina M. Wimo A. G. M. Ali G. C. Wu Y. T. Prina M. (2015). World Alzheimer report 2015: the global impact of dementia: an analysis of prevalence, incidence, cost and trends. Alzheimer's Dis. Int. Available online at: https://www.alzint.org/resource/world-alzheimer-report-2015/

[B45] Ravona-Springer R. Heymann A. Schmeidler J. Guerrero-Berroa E. Sano M. Preiss R.. (2013). Haptoglobin 1-1 genotype is associated with poorer cognitive functioning in the elderly with type 2 diabetes. Diabetes Care 36, 3139–3145. 10.2337/dc12-2250 23990521 PMC3781506

[B46] Rebollido-Rios R. Venton G. Sánchez-Redondo S. Iglesias I. F. C. Fournet G. (2020). Dual disruption of aldehyde dehydrogenases 1 and 3 promotes functional changes in the glutathione redox system and enhances chemosensitivity in nonsmall cell lung cancer. Oncogene 39, 2756–2771. 10.1038/s41388-020-1184-9 32015486 PMC7098886

[B47] Reiman E. M. (2014). Alzheimer's disease and other dementias: advances in 2013. Lancet Neurol. 13, 3–5. 10.1016/S1474-4422(13)70257-6 24331781

[B48] Román G. C. Tatemichi T. K. Erkinjuntti T. Cummings J. L. Masdeu J. C. Garcia J. H.. (1993). Vascular dementia: diagnostic criteria for research studies. Report of the NINDS-AIREN International Workshop. Neurology 43, 250–260. 10.1212/WNL.43.2.250 8094895

[B49] Rosa-Neto P. Hsiung G. Y. Masellis M. (2013). Fluid biomarkers for diagnosing dementia: rationale and the Canadian Consensus on Diagnosis and Treatment of Dementia recommendations for Canadian physicians. Alzheimers. Res. Ther. 5:S8. 10.1186/alzrt223 24565514 PMC3980280

[B50] Sadrzadeh S. M. Bozorgmehr J. (2004). Haptoglobin phenotypes in health and disorders. Am. J. Clin. Pathol. 121(Suppl), S97–104. 10.1309/8GLX5798Y5XHQ0VW 15298155

[B51] Shao C. Zhao M. Chen X. Sun H. Yang Y. Xiao X. (2019). Comprehensive analysis of individual variation in the urinary proteome revealed significant gender differences. Mol. Cell. Proteomics 18, 1110–1122. 10.1074/mcp.RA119.001343 PMC6553935 30894400

[B52] Sutandy F. X. R. Ebersberger S. Huang L. Busch A. Bach M. Kang H. S.. (2018). *In vitro* iCLIP-based modeling uncovers how the splicing factor U2AF2 relies on regulation by cofactors. Genome Res. 28, 699–713. 10.1101/gr.229757.117 29643205 PMC5932610

[B53] Suzuki K. Watanabe T. Sakurai S. Ohtake K. Kinoshita T. Araki A.. (1999). A novel glycosylphosphatidyl inositol-anchored protein on human leukocytes: a possible role for regulation of neutrophil adherence and migration. J. Immunol. 162, 4277–4284. 10201959

[B54] Tollefsen D. M. (2007). Heparin cofactor II modulates the response to vascular injury. Arterioscler. Thromb. Vasc. Biol 27, 454–460. 10.1161/01.ATV.0000256471.22437.88 17194895

[B55] Wang Y. R. Yang Y. H. Lu C. Y. Chen S. H. (2015). Utilization of magnetic nanobeads for analyzing haptoglobin in human plasma as a marker of Alzheimer's disease by capillary electrophoretic immunoassay with laser-induced fluorescence detection. Anal. Chim. Acta 865, 76–82. 10.1016/j.aca.2015.01.030 25732587

[B56] World Health Organization (2017). Global Action Plan on the Public Health Response to Dementia 2017–2025. 52.

[B57] Yang J. L. Mukda S. Chen S. D. (2018). Diverse roles of mitochondria in ischemic stroke. Redox Biol. 16, 263–275. 10.1016/j.redox.2018.03.002 29549824 PMC5854930

[B58] Yerbury J. J. Kumita J. R. Meehan S. Dobson C. M. Wilson M. R. (2009). alpha2-Macroglobulin and haptoglobin suppress amyloid formation by interacting with prefibrillar protein species. J. Biol. Chem. 284, 4246–4254. 10.1074/jbc.M807242200 19074141

[B59] Zhu C. J. Jiang G. X. Chen J. M. Zhou Z. M. Cheng Q. (2018). Serum haptoglobin in Chinese patients with Alzheimer's disease and mild cognitive impairment: a case-control study. Brain Res. Bull. 137, 301–305. 10.1016/j.brainresbull.2018.01.005 29325993

