# Supplementary material for: Label-Free Liquid Chromatography–Mass Spectrometry Proteomic Analysis of Urinary Identification in Diabetic Vascular Dementia in a Han Chinese Population
Source: Front Aging Neurosci. 2021 Feb 1;13:619945. doi: 10.3389/fnagi.2021.619945 (PMC7882624; doi:10.3389/fnagi.2021.619945)
Supplement: Supplementary file 6 [file Table_6.DOCX]

**TABLE S6**  Details of differently expressed proteins identified.

| Entry | Entry name | Protein names | Gene names | Organism | Length |
| --- | --- | --- | --- | --- | --- |
| [P00738](https://www.uniprot.org/uniprot/P00738) | HPT_HUMAN | ****Haptoglobin**** (Zonulin) [Cleaved into: ****Haptoglobin alpha chain****; ****Haptoglobin beta chain****] | ****HP**** | [Homo sapiens (Human)](https://www.uniprot.org/taxonomy/9606) | 406 |
| [P05546](https://www.uniprot.org/uniprot/P05546) | HEP2_HUMAN | ****Heparin cofactor 2**** (Heparin cofactor II, HC-II) (Protease inhibitor leuserpin-2, HLS2) (Serpin D1) | ****SERPIND1**** HCF2 | [Homo sapiens (Human)](https://www.uniprot.org/taxonomy/9606) | 499 |
| [P24539](https://www.uniprot.org/uniprot/P24539) | AT5F1_HUMAN | ****ATP synthase F(0) complex subunit B1, mitochondrial**** (ATP synthase peripheral stalk-membrane subunit b) (ATP synthase proton-transporting mitochondrial F(0) complex subunit B1) (ATP synthase subunit b, ATPase subunit b) | ****ATP5PB**** ATP5F1 | [Homo sapiens (Human)](https://www.uniprot.org/taxonomy/9606) | 256 |
| [O95498](https://www.uniprot.org/uniprot/O95498) | VNN2_HUMAN | ****Vascular non-inflammatory molecule 2, Vanin-2, EC 3.5.1.92****(Glycosylphosphatidyl inositol-anchored protein GPI-80) (Protein FOAP-4) | ****VNN2**** | [Homo sapiens (Human)](https://www.uniprot.org/taxonomy/9606) | 520 |
| Q6PKA6 | AL3A1_HUMAN | ****Aldehyde dehydrogenase, dimeric NADP-preferring, EC 1.2.1.5****(ALDHIII) (Aldehyde dehydrogenase 3) (Aldehyde dehydrogenase family 3 member A1) | ****ALDH3A1****ALDH3 | [Homo sapiens (Human)](https://www.uniprot.org/taxonomy/9606) | 453 |
| B5BU25 | U2AF2_HUMAN | ****Splicing factor U2AF 65 kDa subunit**** (U2 auxiliary factor 65 kDa subunit, hU2AF(65), hU2AF65) (U2 snRNP auxiliary factor large subunit) | ****U2AF2**** U2AF65 | [Homo sapiens (Human)](https://www.uniprot.org/taxonomy/9606) | 475 |
| [P13671](https://www.uniprot.org/uniprot/P13671) | CO6_HUMAN | ****Complement component C6**** | ****C6**** | [Homo sapiens (Human)](https://www.uniprot.org/taxonomy/9606) | 934 |
