# Supplementary material for: Label-Free Liquid Chromatography–Mass Spectrometry Proteomic Analysis of Urinary Identification in Diabetic Vascular Dementia in a Han Chinese Population
Source: Front Aging Neurosci. 2021 Feb 1;13:619945. doi: 10.3389/fnagi.2021.619945 (PMC7882624; doi:10.3389/fnagi.2021.619945)
Supplement: Supplementary file 7 [file Image_1.pdf]

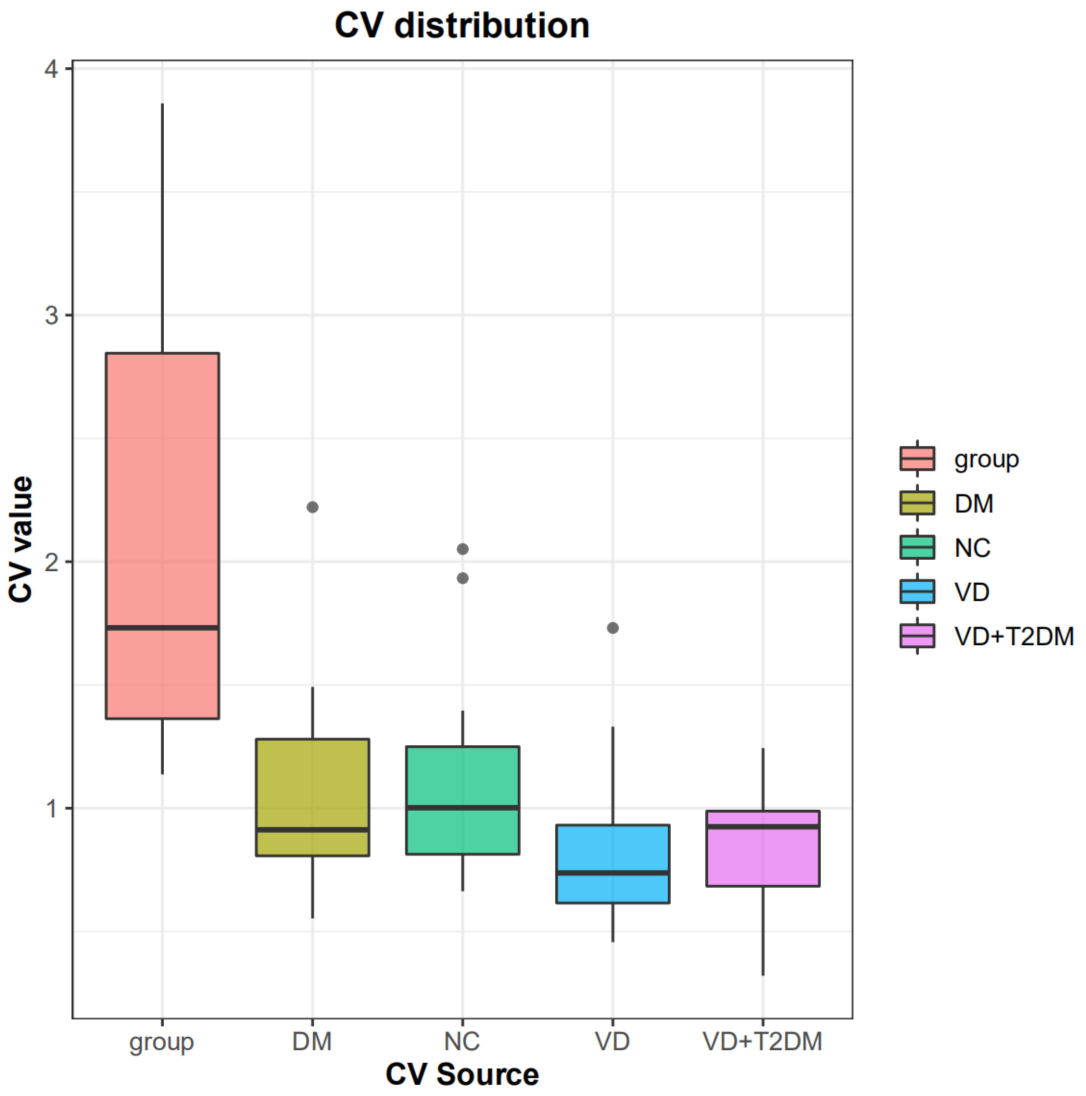

Fig. S1 Inter-individual variations of the urine proteome calculated from 13 urinary proteins(P00738, P05546, P24539, O95498, Q6PKA6, A0A5C2GRG5, A0A5C2FZ29, P07911, Q8N5N7, A5PLM9, B3KY78, A0A384MDZ8, A0A5C2FWX9)
